# Promising Norlabdane-Heterocyclic Hybrids: Synthesis, Structural Characterization and Antimicrobial Activity Evaluation

**DOI:** 10.3390/ph18091411

**Published:** 2025-09-19

**Authors:** Lidia Lungu, Alexandru Ciocarlan, Ionel I. Mangalagiu, Aculina Aricu

**Affiliations:** 1 Institute of Chemistry, Moldova State University, 3 Academiei Street, MD-2028 Chisinau, Moldova; lidilungu@yahoo.com (L.L.); algciocarlan@yahoo.com (A.C.); 2 Faculty of Chemistry, Alexandru Ioan Cuza University of Iasi, 11 Carol Bd., 700506 Iasi, Romania; ionelm@uaic.ro

**Keywords:** norlabdane-heterocyclic hybrids, diazines, 1,2,4-triazoles, 1,3,4-oxadiazoles, 1,3,4-thiadiazoles, 1,3-thiazoles, 1,3-benzothiazoles, 1,3-benzimidazoles

## Abstract

The terpeno-heterocyclic molecular hybrids are a new and promising class of modern organic and medicinal chemistry, because their molecules exhibit high and selective biological activity, natural origins, and good biocompatibility, and, usually, they are less toxic. The reported norlabdane-heterocyclic hybrids were synthesized by classical and new, original, and environmentally friendly methods, which include coupling reactions of norlabdane derivatives (such as carboxylic acids, acyl chlorides, or bromides) with individual heterocyclic compounds, as well as heterocyclization reactions of certain norlabdane intermediates like hydrazides, thiosemicarbazones, or hydrazinecarbothioamides. The aforementioned norlabdanes were derived from (+)-sclareolide **2**, which is readily obtained from (−)-sclareol **1**, a labdane-type diterpenoid extracted from the waste biomass of Clary sage (*Salvia sclarea* L.) that remains after essential oil extraction. All synthesized compounds were tested against various fungal strains and bacterial species, with many exhibiting significant antifungal and antibacterial activity. These findings support the potential application of the synthesized compounds in the treatment of diseases caused by fungi and bacteria. Additionally, the use of plant-based waste materials as starting resources highlights the economic and ecological value of this approach. This review summarizes experimental data on the synthesis and biological activity of norlabdane: diazine, 1,2,4-triazole and carbazole, 1,3,4-oxadiazole, 1,3,4-thiadiazole, 1,3-thiazole, 1,3-benzothiazole and 1,3-benzimidazole hybrids performed by our research group covering the period from 2013 to the present.

## 1. Introduction

The rapid proliferation of microbial infections in recent decades has become a pressing issue in the realm of global public health [1,2]. This trend has driven the search for new molecular structures with antimicrobial properties, aiming to develop effective medicinal agents for their treatment. Among the most promising sources of new biologically active compounds are natural products, due to their biocompatibility, selective biological activity, and typically low toxicity. Terpenes and terpenoids are diverse classes of natural compounds, particularly notable for their wide range of applications in medicine, pharmaceuticals, cosmetics and agriculture [3,4]. Particular attention is paid to terpene compounds, which exhibit multiple biological activities, including anticancer [5], antioxidant, antimicrobial [6], antifungal [7], antimalarial [8], and antidiabetic properties [9]. Some studies have shown that the introduction of heteroatoms, especially nitrogen and sulfur, into terpene structures often enhances their biological activity. Furthermore, the incorporation of heteroatomic functional groups, specific molecular fragments, or heterocyclic units can significantly increase the therapeutic potential of these compounds [10,11].

The synthesis of hybrid molecules—structures that integrate multiple pharmacophores—has emerged as a powerful strategy in drug design. These hybrid compounds often exhibit superior bioactivity compared to existing drugs [12]. In this context, the functionalization of norlabdane derivatives of (−)-sclareol **1**, including sesquiterpenoids (pentanorlabdanes), is of considerable interest. Modifications performed either on the side chain or at the C_7_ position of the B ring have successfully yielded products containing heterocyclic units, paving the way for novel bioactive agents.

**Figure 1:**
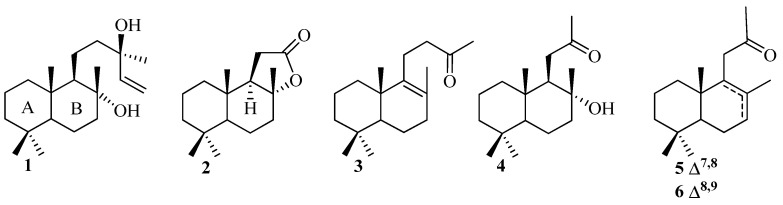
(−)-Sclareol **1**, (+)-sclareolide **2**, their dinorlabdane **3** and trinorlabdane **4**–**6** intermediates.

The main goal of the syntheses presented in this review was the synthesis of norlabdane-heterocyclic molecular hybrids from (−)-sclareol **1**, a readily available natural product that serves as the foundation for these compounds [13,14,15,16,17]. (+)-Sclareolide **2** as an oxidation product of (−)-sclareol **1** [18,19], and its derivatives, such as ketones **3**–**6**, served as starting materials in the syntheses of di- and trinorlabdane-heterocyclic hybrids (Figure 1).

**Figure 2:**
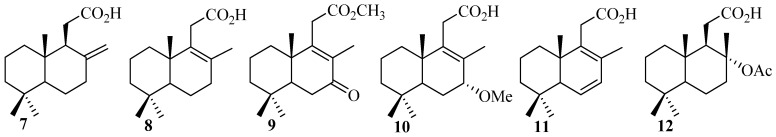
Tetranorlabdane intermediates **7**–**12** of (−)-sclareol **1**.

**Figure 3:**
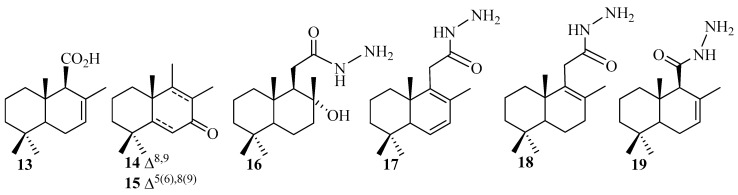
Penta- and tetranorlabdane intermediates and hydrazides **13**–**19** from (−)-sclareol **1**.

In other cases, the syntheses of penta- and tetranorlabdane-heterocyclic hybrids started from carboxylic acids **7**, **8** and **10**–**13** or ketones **9**, **14** and **15**, derived from compounds **1** via (+)-sclareolide **2** (Figure 2 and Figure 3).

Some series of penta- and tetranorlabdane-heterocyclic hybrids were obtained through heterocyclization reactions via hydrazides **16**–**19**, derived from compounds **1** and **2** (Figure 3).

It should be noted that the methods for obtaining the norlabdanic intermediates depicted in Figure 1, Figure 2 and Figure 3 from compounds **1** and **2** are mentioned in the corresponding subchapters.

For convenience, the syntheses of norlabdane: diazine, 1,2,4-triazole and carbazole, 1,3,4-oxadiazole, 1,3,4-thiadiazole, 1,3-thiazole, 1,3-benzothiazole, and 1,3-benzimidazole hybrids are organized into separate Section 2.1, Section 2.2, Section 2.3, Section 2.4, Section 2.5, Section 2.6 and Section 2.7.

## 2. Results

### 2.1. Synthesis of Norlabdane–Diazine Hybrids

Many drugs have been designed based on diazine compounds. Diazines have proven highly valuable in medicine, pharmaceuticals, and cosmetics due to their broad spectrum of biological activities, including antimicrobial, antifungal, antitubercular, antiviral, anti-HIV, and anticancer properties [20]. In this subsection, the synthesis of norlabdane–diazine hybrids will be discussed.

The synthesis of the mentioned hybrids was carried out based on (+)-sclareolide **2**, via ∆^8,13^-bicyclohomofarnesenoic **7**, 13,14,15,16-tetranorlabd-6,8(9)-dien-12-oic **11** and drimenoic **13** acids which were obtained in 5 to 6 steps with total yields of 60%, 94%, and 55%, respectively. Next, the condensation reactions of their acyl chlorides **20**, **21** and **22**, obtained in situ after treatment with oxalyl chloride in anhydrous benzene, with corresponding amines **23a**–**c** (2-aminopyrimidine, 2-aminopyrazine and 4-aminopyrimidine) were performed [21].

**Scheme 1:**
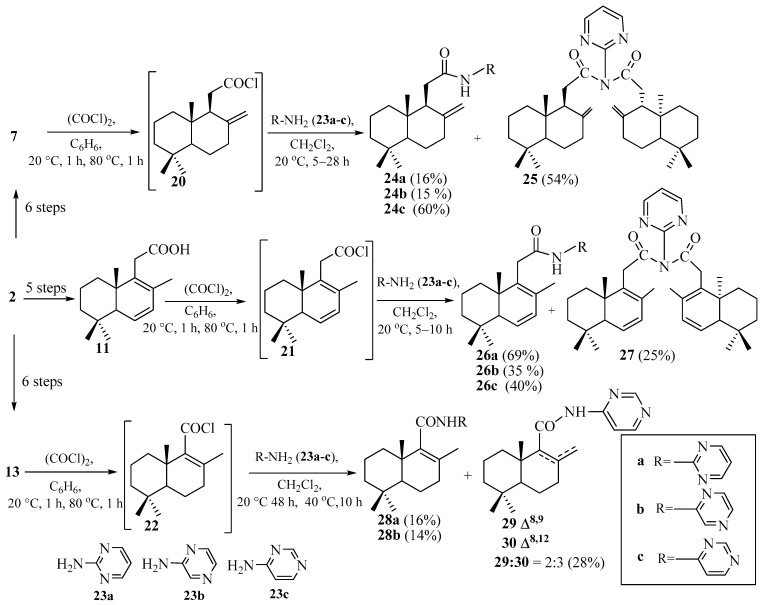
Synthesis of norlabdane–diazine hybrids. Data from [21,22,23,24].

As a result, from bicyclohomofarnesenoyl chloride **20** a series of new hybrid amides **24a**–**c** and **25** were obtained (Scheme 1) [21,22]. It should be noted that in the case of 2-aminopyrazine **23b** and 4-aminopyrimidine **23c**, only monoacylamides **24b** (15%) and **24c** (60%) were formed, while for 2-aminopyrimidine **23a**, in addition to monoacylamide **24a** (16%), *bis*-acylamide **25** (54%) was also produced.

The molecular structure of hybrid **25** was established spectrally and its stereochemistry was confirmed based on single-crystal X-ray diffraction. It was determined that the *bis*-acylation process occurs only in the case of 2-aminopyrimidine.

The coupling reactions of 13,14,15,16-tetranorlabd-6,8(9)-dien-12-oyl chloride **21** with amides **23a**–**c** were carried out under the same conditions, in methylene chloride while stirring. In the case of 2-aminopyrazine **23b** and 4-aminopyrimidine **23c**, only monoacylamides **26b** and **26c** were obtained, with yields of 35% and 40%, respectively (Scheme 1). In the case of 2-aminopyrimidine **23a**, two compounds were similarly obtained: monoacylamide **26a** (69%) and *bis*-acylamide **27** (25%) [23].

All *N*-substituted amides, derived both from Δ^8,13^-bicyclohomofarnesenoic acid **7** and from 13,14,15,16-tetranorlabd-6,8(9)-dien-12-oic acid **11**, are obtained through the condensation of primary amines with acyl chlorides **20** and **21**. Secondary amides are also capable of undergoing further reaction with acyl chlorides; however, experimental data indicate that only the monoacylamides **24a** and **26a** undergo *bis*-acylation, affording the corresponding *bis*-acylamides **25** and **27**.

This selectivity is likely attributable to the delocalization of the nonbonding electrons of nitrogen into the adjacent carbonyl group (amide bond resonance), which decreases the overall nucleophilicity of the amide nitrogen and thereby reduces its reactivity toward further acylation. Moreover, in the case of amides **24a**–**c** and **26a**–**c**, additional resonance delocalization into the aromatic substituents further modulates their electronic environment, suggesting that the aryl groups exert a pronounced influence on the observed reactivity. Reaction time may also play a critical role in enabling the formation of the *bis*-acylated derivatives.

Drimenoic acid **13** was synthesized from (+)-sclareolide **2** in six steps, with a total yield of 55%, according to the method developed by the authors [24].

Further synthetic efforts were directed toward the preparation of amides of drimenoic acid **13**. Unexpectedly, instead of the anticipated products, isodrimenoic acid amides **28a** and **28b** were isolated in yields of 16% and 14%, respectively. In addition, the reaction of the intermediate acid chloride **22** with 4-aminopyrimidine **23c** furnished a mixture of amides **29** and **30** in a 2:3 ratio, with a total yield of 28% [24].

It should be noted that during the reaction of drimenoic acid **13** with oxalyl chloride, the isomerization of the double bond ∆^7,8^ in the position ∆^8,9^, occurs. As a result, the subsequent reaction of acyl chloride **22** with 2-aminopyrimidine **23a**, 2-aminopyrazine **23b**, and 4-aminopyrimidine **23c** affords the isodrimenoic acid amides **28a**, **28b**, and **29**, as well as the albicanoic acid amide **30**.

The in vitro antimicrobial activities of the synthesized homodrimane sesquiterpenoids, both with and without a diazine skeleton, were systematically evaluated. The tested compounds exhibit excellent antibacterial activity against Gram-positive strains of *S. aureus* and *B. cereus*. Structure-activity relationship correlations reveal that homodrimane sesquiterpenoids with diazine skeleton possess a better antibacterial activity compared to those without a diazine skeleton. Additionally, in the homodrimane sesquiterpenoids with a diazine skeleton series, the 2-aminopyrimidine **23a** derivatives demonstrate better activity than the 2-aminopyrazine **23b** ones and, the mono-acyl amides show better activity than the *bis*-acyl amides. Compounds **24a**–**c** and **25** from this series exhibited good antibacterial activity, but none of the tested compounds showed antifungal activity [22].

As a continuation of previous research on the synthesis of new compounds incorporating both terpene and 1,2-diazine fragments, a series of norlabdane derivatives containing a pyridazinone structural unit were synthesized [25,26].

For the preparation of these norlabdane-based compounds, the methyl ester of 7-oxo-13,14,15,16-tetranorlabd-8-en-12-oic acid **9** was used as the starting material. This intermediate can be obtained from (+)-sclareolide **2** in two steps, with an overall yield of 77%.

**Scheme 2:**
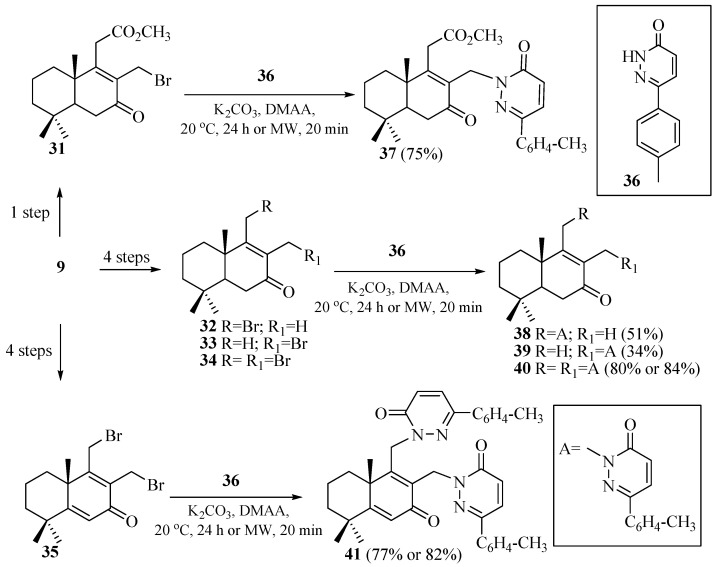
Synthesis of tetra- and pentanorlabdane-*p*-tolyl-pyridazinone hybrids. Data from [25,26,27].

The ketoester **9** was subsequently brominated using *N*-bromosuccinimide (NBS, 1.5 equivalents), resulting in the formation of the corresponding tetranorlabdane bromide **31** (Scheme 2).

Subsequently, ketobromide **31** was coupled with 6-(*p*-tolyl)-3(2*H*)-pyridazinone **36** in a basic medium (K_2_CO_3_) using *N,N*-dimethylacetamide (DMAA) as the solvent, leading to the formation of the tetranorlabdane–pyridazinone hybrid compound **37**. This reaction proceeds efficiently due to the conjugated unsaturated nature of pyridazinone **36**, which enhances the acidity of the *N*–H bond, making it readily deprotonated under basic conditions (Scheme 2). The coupling was conducted under both conventional thermal conditions and under microwave irradiation [25].

Next, through a series of transformations starting from the methyl ester of 7-oxo-13,14,15,16-tetranorlabd-8-en-12-oic acid **9**, two pentanorlabdane ketone intermediates were obtained. These ketones were then treated with *N*-bromosuccinimide (NBS) in carbon tetrachloride, leading to the formation of several brominated derivatives: 11-bromo-drim-8(9)-en-7-one **32**, 12-bromo-drim-8(9)-en-7-one **33**, 11,12-dibromo-drim-8(9)-en-7-one **34**, and 11,12-dibromo-drim-5(6),8(9)-dien-7-one **35**. The ratio of these brominated products in the reaction mixture can be adjusted by altering the amount of NBS and the reaction time (Scheme 2).

The brominated compounds **32**–**35** were subsequently subjected to coupling reactions with 6-(*p*-tolyl)-3(2*H*)-pyridazinone **36** in a basic medium (KOH) using *N,N*-dimethylacetamide (DMAA) as the solvent. These reactions were carried out under both conventional heating and microwave irradiation conditions. A chromatographically inseparable mixture of bromides **32** and **33** (in a 3:2 ratio) was reacted with 6-(*p*-tolyl)-3(2*H*)-pyridazinone **36**, yielding the monosubstituted hybrids 11-*p*-tolyl-pyridazinyl-drim-8(9)-en-7-one **38** and 12-*p*-tolyl-pyridazinyl-drim-8(9)-en-7-one **39**. Unlike the starting bromides, these products could be separated by silica gel column chromatography. The combined overall yield of compounds **38** and **39** was 85%. Individual dibromides **34** and **35**, when treated with pyridazinone **36** under the same reaction conditions, produced 11,12-di(*p*-tolyl-pyridazinyl)-drim-8(9)-en-7-one **40** and 11,12-di(*p*-tolyl-pyridazinyl)-drim-5(6),8(9)-dien-7-one **41**, respectively.

The structures of the newly synthesized compounds were confirmed using spectroscopic methods, including ^1^H and ^13^C NMR, and IR spectroscopy. Additionally, the stereochemistry of compound **41** was established by single-crystal X-ray diffraction analysis [26].

All synthesized compounds were evaluated in vitro against five fungal strains and two bacterial species.

Compound **41**, featuring a quinone-analogue skeleton in combination with two diazine units, exhibits high antibacterial and antifungal activity. Its antifungal activity was recorded at an MIC of 5 × 10^−3^ μg/mL, while its antibacterial activity (MIC = 3.2 × 10^−2^ μg/mL) was approximately 90 times more active than Kanamycin (3 μg/mL) [27]. The enhanced activity of compound **41** can be attributed to the synergistic effect of the quinone moiety, which is known to participate in redox cycling and disrupt microbial metabolic pathways, together with the two diazine rings that may improve molecular interactions with biological targets.

Compound **39**, which includes only a diazine ring at C_12_ of the norlabdane core and lacks the analogous quinone moiety, exhibits moderate antifungal activity (MIC = 15 × 10^−1^ μg/mL), but it is six times lower compared to the reference compound Caspofungin (0.25 μg/mL). Compounds **38** and **40** do not exhibit inhibitory activity against the aforementioned strains of bacteria and fungi.

Thus, for the first time, based on (+)-sclareolide **2**, syntheses of penta- and tetranorlabdane–diazine hybrids were achieved, one of which exhibited exceptional antimicrobial activity that was patented [27].

### 2.2. Synthesis of Norlabdane-1,2,4-triazole and Carbazole Hybrids

In the previous subchapter, molecular hybridization methods applied to obtain homodrimane sesquiterpenoids with diazine units were described [21]. Although none of the hybrids depicted in Scheme 1 were active against fungi, some demonstrated high antibacterial activity [22]. This fact directed the attention of our research team toward other heterocyclic units suitable for molecular hybridization, such as 1,2,4-triazole units.

This structural core is widely represented in both natural products and therapeutic agents, and its substituted derivatives are regarded as privileged pharmacophores in compounds with anticancer, antimicrobial, and antiviral activities [28,29,30,31].

The first attempt to obtain *N*-substituted tetranorlabdane-1,2,4-triazole hybrids was made by the authors [32]. The products obtained resulted in a substantial increase in the antioxidant activity of the biomass of certain cyanobacterial species, which were patented together along with cultivation methods [33].

Next, the results of the syntheses of some norlabdane hybrids with 1,2,4-triazole units via the corresponding acyl chlorides and hydrazinecarbothioamides, the structures and biological properties of which have been elucidated, will be reported.

**Scheme 3:**
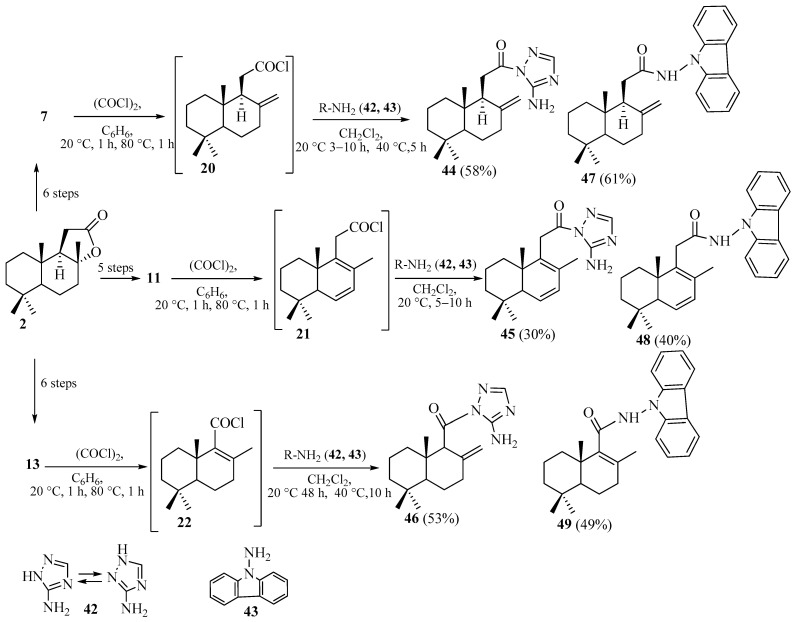
Synthesis of norlabdane-1,2,4-triazole and carbazole hybrids. Data from [23,24,32].

The synthesis of amides **44** and **47** of ∆^8,13^-bicyclohomofarnesenoic acid **7** and amides **45** and **48** of 13,14,15,16-tetranorlabd-6,8(9)-dien-12-oic acid **11**, which include 1,2,4-triazole and carbazole rings, was carried out according to Scheme 3 [23,24].

Upon interaction of acids **7** and **11** with (COCl)_2_, the acyl chlorides **20** and **21** were obtained in situ. As a result of their reaction with 3-amino-1,2,4-triazole **42** and *N*-aminocarbazole **43**, the amides **44**, **45**, **47** and **48** were formed. In the case of drimenoic acid **13**, contrary to expectations, albicanoic acid **46** and isodrimenoic acid **49** amides were obtained [24]. It should be noted that under the conditions of the reaction of drimenoic acid **13** with oxalyl chloride, isomerization of the ∆^7,8^ double bond to the ∆^8,9^ position occurs, which leads to the subsequent interaction of the acyl chloride **22** with *N*-aminocarbazole **43** forming albicanoic acid **46** and isodrimenoic acid **49** amides. Spectral analysis of albicanoic acid amide **46** revealed that 3-amino-1,2,4-triazole **42** participated in the reaction in its tautomeric form, resulting in the incorporation of an amino group and the preservation of the semicyclic double bond Δ^8,13^ within the amide structure.

The structure and stereochemical configuration of *N*-(isodrimenoylamino)carbazole **49** were unambiguously established by single-crystal X-ray diffraction analysis.

In the continuation of the research in the field of norlabdane compounds with biologically active heterocyclic fragments, a series of norlabdane-1,2,4-triazole hybrids was synthesized by an alternative method [34].

**Scheme 4:**
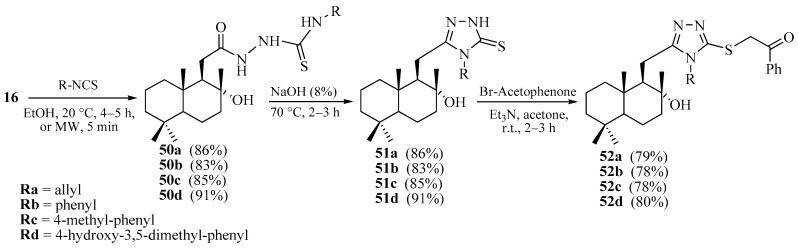
Synthesis of norlabdane-1,2,4-triazole hybrids. Data from [34].

For the preparation of the aforementioned derivatives, 8*α*-hydroxy-homodrim-11-hydrazide **16** was employed as the key starting material. This compound was obtained in a single step from commercially available (+)-sclareolide **2** with a yield of 85%, as illustrated in Scheme 4 [34]. Next, hydrazide **16** was coupled with substituted aryl isothiocyanates in ethanol, resulting in hydrazinecarbothioamides **50a**–**d**. The synthesis of hydrazine carbothioamides **50a**–**d** by the classical method was carried out at room temperature for 270–300 min, which has a disadvantage. For these reasons, unconventional methods were used, and the synthesis was carried out by microwave irradiation at a constant power of 200 W. According to the experimental data obtained from microwave irradiation, the reaction time decreases considerably (from several hours to 5 min), and the yields increase slightly. Therefore, the coupling reactions of hydrazide **16** with aryl isothiocyanates through microwave irradiation are considered environmentally friendly [34].

Hydrazine carbothioamides **50a**–**d** were treated with aqueous NaOH (8%) at 70 ˚C to afford the corresponding norlabdane derivatives bearing a triazole moiety **51a**–**d** in yields of 70–83%. Subsequent coupling of triazoles **51a**–**d** with bromoacetophenone in acetone, in the presence of triethylamine (Et_3_N), furnished the *S*-substituted 1,2,4-triazoles **52a**–**d**.

Theoretically, the *N*–H functional group of the triazole moiety can undergo condensation with a halogenated aromatic derivative such as bromoacetophenone. However, in compounds **51a**–**d** the triazole ring exists in two resonance forms, one of which corresponds to an aromatic zwitterion with a positively charged N1 and a negatively charged sulfur atom. The excess electron density on sulfur renders it nucleophilic, enabling preferential reaction with bromoacetophenone to give the *S*-substituted products.

The structures of these compounds were elucidated on the basis of spectral data (^1^H and ^13^C NMR, IR), while the stereochemistry of compounds **51c** and **51d** was unambiguously confirmed by single-crystal X-ray diffraction.

The antibacterial and antifungal activities of the synthesized compounds with hydrazide **16**, hybrid norlabdane and carbothioamide **50a**–**d**, or triazole skeletons **51a**–**d** and **52a**–**d** were tested in vitro on pure cultures of bacteria and fungi.

Compounds **51c** and **51d** displayed remarkable antimicrobial activities, with MIC values of 0.125 μg/mL and 0.094 μg/mL for antifungal activity and 0.064 μg/mL and 0.047 μg/mL for antibacterial activity, respectively. Notably, the antibacterial potency of triazole **51d** was 63-fold greater than that of the reference antibiotic Kanamycin (MIC 3.5 μg/mL), while its antifungal effect was threefold stronger than that of Caspofungin (MIC 0.24 μg/mL). In contrast, the *S*-substituted triazoles **52a**–**d**, in which the thiol group at position 2 of the triazole ring is replaced by an acetophenone moiety, were devoid of antimicrobial activity.

In addition, tetranorlabdane hydrazinecarbothioamides **50c** and **50d** were subjected to cytotoxicity testing on human ovarian carcinoma cell lines A2780 and A2780cis, as well as on the non-cancerous human renal embryonic cell line HEK293 [34], both demonstrating moderate activity in the micromolar IC_50_ range.

Thus, for the first time, based on (+)-sclareolide **2**, efficient syntheses of biologically active homodrimane hybrids with hydrazinecarbothioamide fragments or 1,2,4-triazole and carbazole rings have been achieved with high yields, both by classical methods and by microwave irradiation.

### 2.3. Synthesis of Norlabdane-1,3,4-oxadiazole Hybrids

1,3,4-Oxadiazole is a versatile heterocyclic ring, frequently used in pharmaceutical chemistry for the development of novel therapeutic agents [35]. Various 1,3,4-oxadiazole derivatives have attracted significant interest due to their broad range of biological activities, including antimitotic, antifungal, antibacterial, sedative-hypnotic and anticonvulsant activities among others [36,37,38,39]. Due to their great medical significance, numerous synthetic routes for 1,3,4-oxadiazoles have been developed, some of which have been described by the authors [40].

Next, the synthesis and biological properties of new molecular norlabdane-1,3,4-oxadiazole hybrids will be described [41], which is a continuation of our research in the field of compounds with cumulative biological potential [22,26,32].

The synthesis of the reported compounds was carried out based on 8*α*-hydroxy-homodrim-11-hydrazide **16**, previously obtained from commercial (+)-sclareolide **2** in one step with a yield of 85% [41].

**Scheme 5:**
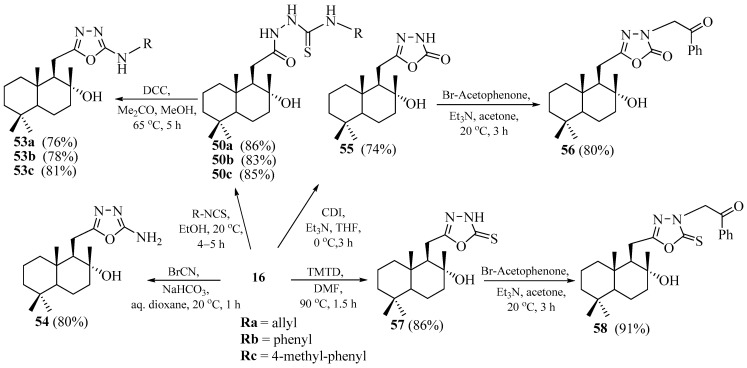
Synthesis of tetranorlabdane-1,3,4-oxadiazole hybrids. Data from [41].

Treatment of hydrazide **16** with substituted aryl isothiocyanates in EtOH afforded the corresponding hydrazinecarbothioamides **50a**–**c** in 83–86% yields, as shown in Scheme 5 [34].

Being treated with *N*,*N*′-dicyclohexylcarbodiimide (DCC) in a mixture of MeOH and Me_2_CO [42], carbothioamides **50a**–**c** formed tetranorlabdane hybrids with substituted 2-amino-1,3,4-oxadiazole units **53a**–**c** in 76–81% yields.

Next, hydrazide **16** was treated with cyanogen bromide (BrCN) in aqueous dioxane [43], yielding unsubstituted 5-(8α-hydroxydriman-11-yl)-1,3,4-oxadiazol-2-amine **54** in 80% yield.

The reaction of hydrazide **16** with 1,1′-carbonyldiimidazole (CDI) in tetrahydrofuran (THF) in the presence of triethylamine led to 5-(8*α*-hydroxydriman-11-yl)-1,3,4-oxadiazol-2(3*H*)-one **55** in 74% yield according to Scheme 5 [43].

A study was conducted on the reaction between hydrazide **16** and varying amounts of tetramethylthiuram disulfide (TMTD), by heating at 90 °C in dimethylformamide (DMF) according to the known procedure [44].

The use of 1 equivalent of TMTD under the mentioned conditions proved to be quite efficient and led to 2-thio-5-(11-homodrim-8*α*-ol)-1,3,4-oxadiazole **57** in 86% yield and a small amount of a 1,3,4-thiadiazole hybrid which will be described in Section 2.4 [41].

Next, 1,3,4-oxadiazoles **55** and **57** were subjected to coupling reactions with bromoacetophenone in acetone in the presence of Et_3_N to form 3-*N*-acetophenone-5-(11-homodrim-8*α*-ol)-1,3,4-oxadiazol-2-one **56** in 80% yield and 3-*N*-acetophenone-5-(11-homodrim-8*α*-ol)-1,3,4-oxadiazole-2-thione **58** in a 91% yield (Scheme 5) [45].

**Scheme 6:**
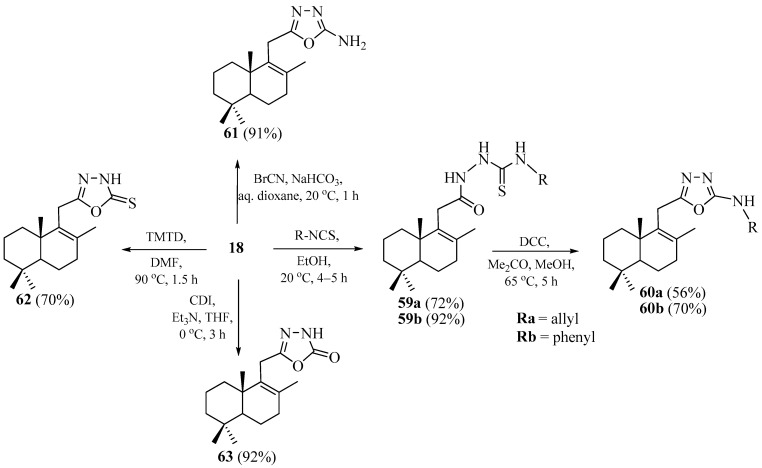
Synthesis of tetranorlabdane-1,2,4-oxadiazole hybrids. Data from [46].

For the preparation of tetranorlabdane-1,3,4-oxadiazole hybrids, hydrazide **18** of Δ^8^,^9^-bicyclohomofarnesenoic acid was used as the starting material. This compound was synthesized from commercially available (+)-sclareolide **2** in seven steps, affording an overall yield of 40% [46] (Scheme 6).

The reaction of hydrazide **18** with allyl or phenyl isothiocyanate in ethanol led to the formation of hydrazine carbothioamides **59a** and **59b**, which subsequently, upon treatment with *N*,*N*′-dicyclohexylcarbodiimide (DCC) at reflux in methanol, are transformed into allylamino- and phenylamino 1,3,4-oxadiazole derivatives **60a** and **60b** substituted in position 2, in depicted yields (Scheme 6).

Following the interaction of hydrazide **18** with cyanogen bromide (BrCN) in aqueous dioxane, 2-amino-5-(Δ^8,9^-bicyclohomofarnesenyl)-1,3,4-oxadiazole **61** was obtained with a yield of 91%.

The 2-thio-5-(Δ^8,9^-bicyclohomofarnesenyl)-1,3,4-oxadiazole **62** was prepared under standard conditions by heating hydrazide **18** at 90 °C in dimethylformamide (DMF) with tetramethylthiuram disulfide (TMTD) in 70% yield.

The reaction of hydrazide **18** with 1,1′-carbonyldiimidazole (CDI) in tetrahydrofuran (THF) in the presence of triethylamine led to 5-(Δ^8,9^-bicyclohomofarnesenyl)-1,3,4-oxadiazole-2(3*H*)-yl **63** in 92% yield (Scheme 6).

**Scheme 7:**
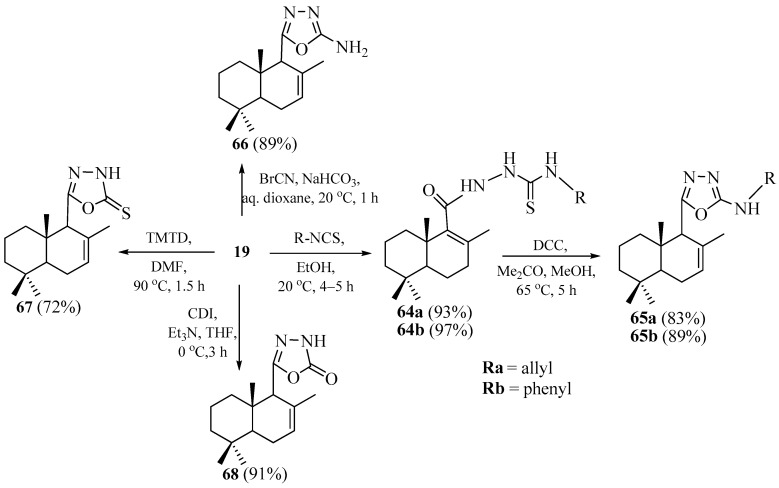
Synthesis of pentanorlabdane-1,2,4-oxadiazole hybrids. Data from [46].

To make a comparative study of the structure-activity relationship (SAR) with tetranorlandane molecular hybrids, we synthesized a series of pentanorlabdane 1,3,4-oxadiazoles, starting from the hydrazide of drimenoic acid **18**, prepared according to standard procedure that includes the in situ preparation of drimenoic acid **13** acyl chloride, followed by its interaction with hydrazine hydrate (N_2_H_4_∙H_2_O) (Scheme 7).

Subsequently, starting from hydrazide **19**, under the conditions described above, a series of novel pentanorlabdane 1,3,4-oxadiazole hybrids **65a** (83%) and **65b** (89%), **66** (89%), **67** (72%) and **68** (91%) were obtained (Scheme 7).

Spectral analyses (^1^H and ^13^C NMR, IR) were employed to establish the structures of the synthesized compounds, and the structure as well as the stereochemistry of compound **67** were further validated by sin64gle-crystal X-ray diffraction.

The antibacterial and antifungal activities of the synthesized tetranorlandane **53**–**58**, **59**–**63** and pentanorlabdane **64**–**68** 1,3,4-oxadiazole hybrids were tested in vitro on pure cultures of bacteria and fungi.

According to these, 1,3,4-oxadiazoles **56**, **57** and **63** exhibit significant antifungal activity at a minimum inhibitory concentration (MIC) of 2 μg/mL, 1.3 μg/mL and 0.125 μg/mL, respectively, compared to the reference compound Caspofungin (MIC 0.24 μg/mL). Compound **56** also exhibits pronounced antibacterial activity with an MIC of 0.50 μg/mL, which is seven times higher compared to the reference compound, Kanamycin (MIC 3.5 μg/mL).

Thus, for the first time, based on (+)-sclareolide **2**, syntheses of corresponding 1,3,4-oxadiazole hybrids were achieved through its tetra- and pentanorlabdanic derivatives. By varying the reagents and molecular ratios, the optimal conditions for the heterocyclization reactions were established. All the synthesized compounds were tested in vitro, and five of them showed antimicrobial activity.

### 2.4. Synthesis of Norlabdane-1,3,4-thiadiazole Hybrids

1,3,4-Thiadiazole, as well as its derivatives, contains a universal heterocyclic nucleus that has attracted increased attention in medicinal chemistry in the search for new therapeutic agents. This five-membered heterocyclic compound is widely used as a structural element in various biologically active molecules, including drugs [47], because 1,3,4-thiadiazole derivatives have a broad spectrum of biological activity, including antitumor, antibacterial, antifungal, antituberculosis, anti-inflammatory, antiviral and antileishmanial activities [48].

**Scheme 8:**
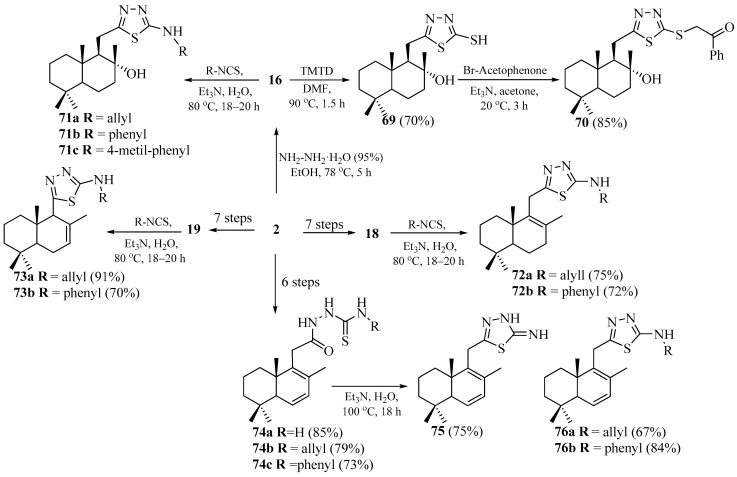
Synthesis of tetra- and pentanorlabdane 1,3,4-thiadiazole hybrids. Data from [41,46,49].

As a starting material for the synthesis of the reported norlabdane 1,3,4-thiadiazoles, 8α-hydroxy-homodrim-11-hydrazide **16** was also used (Scheme 8) [41].

As mentioned in Section 2.3, the reaction of 8*α*-hydroxy-homodrim-11-hydrazide **16** tetramethylthiuram disulfide (TMTD) in DMF yields a mixture of two compounds: 2-thio-5-(11-homodrim-8*α*-ol)-1,3,4-oxadiazole **57** and 2-mercapto-5-(11-homodrim-8*α*-ol)-1,3,4-thiadiazole **69** (Scheme 5 and Scheme 8). The ratio of oxadiazole **57** to thiadiazole **69** hybrids is strongly dependent on the stoichiometry of TMTD used in the reaction [41].

When 0.5 mol of TMTD is used in the reaction with 1 mol of hydrazide **16** only 46% of the initial hydrazide reacts, forming only 1,3,4-oxadiazole **57**. Using equimolar amounts of hydrazide **16** and TMTD produces a mixture of oxadiazole **57** and thiadiazole **69**, with oxadiazole **57** predominating. Increasing the TMTD to 1.5 mol relative to 1 mol of hydrazide **16** results in the same product mixture, but with thiadiazole **69** prevailing (70%). The formation of 1,3,4-thiadiazole **69** was unexpected; however, its structure was fully confirmed by spectral analysis, and the stereochemistry of compound **69** was definitively established by single-crystal X-ray diffraction.

Next, thiadiazole **69** was subjected to coupling reactions with bromoacetophenone in acetone in the presence of Et_3_N to form 2-*S*-acetophenone-5-(11-homodrim-8α-ol)-1,3,4-thiadiazole **70** in 85% yield (Scheme 8).

Following the strategy for synthesizing norlabdane 1,3,4-thiadiazole hybrids, another synthetic method was employed, involving the interaction of hydrazide **16** with isothiocyanate derivatives, without isolating intermediate compounds. This reaction was carried out in the presence of triethylamine (Et_3_N) in water, resulting in 2-amino-1,3,4-thiadiazoles **71a**–**c** with yields of 70–78% (Scheme 8) [41].

The antibacterial and antifungal activity of the synthesized norlabdane 1,3,4-thiadiazole hybrids **69**, **70** and **71a**–**c** was tested in vitro on pure cultures of bacteria and fungi. Thiadiazole **69** exhibits pronounced antifungal activity (at MIC 0.032 μg/mL) and antibacterial activity (at 0.094 μg/mL) [41]. The antibacterial activity of compound **69** is thirty times higher than that of the reference compound, Kanamycin (MIC 3.5 μg/mL). As an antifungal agent this compound is eight times more active than the reference compound Caspofungin (MIC 0.24 μg/mL). Thiadiazole **71a** exhibits antifungal activity (MIC 0.25 μg/mL) and pronounced antibacterial activity (MIC 0.5 μg/mL). The antibacterial activity of compound **71a** is six times higher compared to the reference compound, Kanamycin (MIC 3.5 μg/mL).

Prolonged reflux of hydrazides **18** and **19** with allyl isothiocyanate or phenyl isothiocyanate in water in the presence of Et_3_N afforded tetra- and pentanorlabdane 1,3,4-thiadiazole hybrids, **72a** and **72b**, **73a** and **73b** [41] (Scheme 8).

For the synthesis of novel tetranorlabdane derivatives bearing thiosemicarbazone fragments or 1,3,4-thiadiazole rings, 13,14,15,16-tetranorlabd-6(7),8(9)-dien-12-oic acid **11** was employed as the starting material. This acid was obtained from commercially available (+)-sclareolide (2) in five steps, with an overall yield of 47% [49]. Subsequently, the coupling of acid **11** with allyl- or phenylthiosemicarbazides (molar ratio 1:1.2) was carried out in dichloromethane using EDCI as the coupling reagent, affording the tetranorlabdane thiosemicarbazone derivatives **74a**–**c** in 73–85% yields (Scheme 8).

In continuation, the heterocyclization reaction of thiosemicarbazones **74a**–**c** was carried out in the presence of Et_3_N and H_2_O, yielding the tetranorlabdane 1,3,4-thiadiazole hybrids **75** and **76a**,**b** in 75% and 67–84% yields, respectively (Scheme 8).

The antibacterial and antifungal activities of the synthesized tetranorlabdane hybrids with thiosemicarbazone or 1,3,4-thiadiazole moieties were tested in vitro on pure cultures of bacteria and fungi [50].

Thus, for the first time, based on (+)-sclareolide **2**, through its tetra- and pentanorlabdanic derivatives, syntheses of hybrids with 1,3,4-thiadiazole units were achieved and the mechanism of formation of some compounds was explained. In vitro tests revealed the increased activity of hybrids **69** and **75** and which are of interest to the pharmaceutical industry, and the activity of compounds **69** and **75** was patented [51,52].

### 2.5. Synthesis of Norlabdane-1,3-thiazole Hybrids

The 1,3-thiazole moiety represents a key structural element in drug design due to its wide-ranging biological activities. Several thiazole derivatives are known to exhibit anticonvulsant, antimicrobial, anti-inflammatory, antitumor, and other pharmacologically relevant effects [53,54,55,56]. Similarly, compounds containing thiosemicarbazone fragments display a broad spectrum of biological activities, including antitumor, antifungal, antibacterial, antiviral, and antimalarial properties, etc. [57,58,59], and frequently serve as intermediates in the synthesis of compounds with 1,3-thiazole moieties. Unfortunately, there are few mentions in the specialized literature regarding the synthesis of terpenes with 1,3-thiazole units and the evaluation of their biological activity [60,61,62,63].

In continuation, the data regarding the synthesis of tetra- and pentanorlabdane compounds with thiosemicarbazone moieties and 1,3-thiazole units in outside chain or cycle B synthesized by our group will be described [63].

**Scheme 9:**
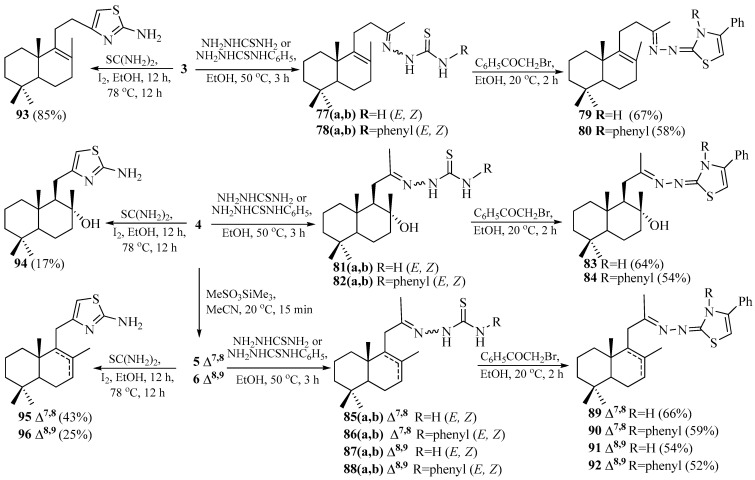
Synthesis of di- and trinorlabdane-1,3-thiazole hybrids. Data from [63,66].

Natural diol (−)-sclareol **1** was used as the starting material for the synthesis of norlabdanic intermediates. Initially, in two consecutive stages it was transformed into unsaturated ketone 15,16-dinorlabd-8(9)-en-13-one **3** in 80% yield (Scheme 9) [64].

The reaction of ketone **3** with thiosemicarbazide or 4-phenylthiosemicarbazide (molar ratio 1:1.1) afforded dinorlabdane compounds with thiosemicarbazone moieties **77** and **78**, each as a mixture of two chromatographically inseparable isomers. The reaction of thiosemicarbazones **77** and **78** with 2-bromoacetophenone in ethanol (molar ratio 1:1) led to the formation of dinorlabdane-1,3-thiazole hybrids **79** and **80**.

For the synthesis of trinorlabdane-1,3-thiazole hybrids, commercially available (+)-sclareolide **2** was used as a starting material. Its interaction with methyllithium (CH_3_Li) gave 8*α*-hydroxy-14,15,16-trinorlab-12-one **4** in 65% yield according to the methodology [64]. Treatment of hydroxyketone **4** with MeSO_3_SiMe_3_ in acetonitrile resulted in a mixture of known 14,15,16-trinorlabd-7(8)-en-13-one **5** and 14,15,16-trinorlab-8(9)-en-13-one **6**, (ratio 4:1), with a 91% overall yield, which were successfully separated by column chromatography on silica gel (Scheme 9) [65].

The reaction of ketones **4**–**6** with thiosemicarbazide or 4-phenylthiosemicarbazide (molar ratio 1:1.1) gave trinorlabdane compounds with thiosemicarbazone moieties **81**, **82** and **85**–**88**. Each of these thiosemicarbazones was obtained as a chromatographically inseparable mixture of two isomers. It should be noted that in subsequent reactions, mixtures of isomeric thiosemicarbazones were used, as it is known that over time, the *Z*-isomers transform into the more stable *E*-isomers. The reaction of thiosemicarbazones **81**, **82** and **85**–**88** with 2-bromoacetophenone in ethanol (molar ratio 1:1) led to the formation of trinorlabdane-1,3-thiazole hybrids **83**, **84** and **89**–**92**.

Further, ketones **3**–**6** underwent a condensation-cyclization reaction with thiourea and iodine in ethanol, to afford the corresponding norlabdane-2-amino-1,3-thiazole hybrids. The unsaturated dinorlabdane ketone **3** yielded only the mentioned 2-amino-4-(15,16-dinorlabd-8(9)-en-13-one)-1,3-thiazole **93** with an overall yield of 85%. In the case of trinorlabdane hydroxyketone **4**, a mixture of thiazoles **94**–**96**, in a ratio of 1:1.5:2.5 was obtained, with an overall yield of 85%. The formation of this mixture may be explained as hydroxyketone **4** undergoes partial dehydration, which leads to 2-amino-4-(14,15,16-trinorlabd-7(8)-en-13-one)-1,3-thiazole **95** and 2-amino-4-(14,15,16-trinorlabd-8(9)-en-13-one)-1,3-thiazole **96**, obtained in 43% and 25% yields, respectively. This fact is confirmed by the formation of minor hydroxylated 2-amino-4-(8*α*-hydroxy-14,15,16-trinorlabd-13-one)-1,3-thiazole **94**, isolated from the reaction mixture in a 17% yield. The condensation-cyclization reaction of unsaturated ketones **5** and **6**, under the same conditions, led to trisubstituted **95** and tetrasubstituted **96** trinorlabdane-1,3-thiazoles [66].

**Scheme 10:**
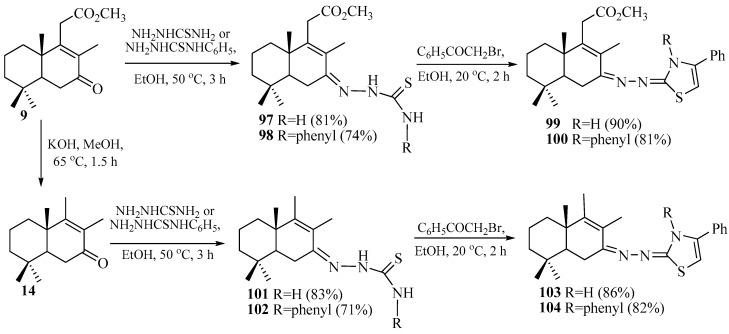
Synthesis of tetra- and pentanorlabdane-1,3-thiazole hybrids. Data from [63].

As a starting material, for the synthesis of cycle B tetra- and pentanorlabdane-1,3-thiazole hybrids, the methyl ester of 7-oxo-13,14,15,16-tetranorlabd-8-en-12-oic acid **9** was used, which was obtained from (+)-sclareolide **2**, in two steps and with a total yield of 76%. Decarboxylation of ketoester **9** was carried out at reflux for 3 h in an alcoholic potassium hydroxide solution, leading to the reference pentanorlabdane intermediate drim-8(9)-en-7-one **14** with a 98% yield (Scheme 10) [67].

Subsequently, in the reaction of ketones **9** and **14** with thio- or 4-phenylthiosemicarbazide in a molar ratio of 1:1.1, tetra- and pentanorlabdane compounds with thiosemicarbazide moieties **97**, **98,** and **101**, **102** were obtained [63,68].

Tetra- and pentanorlabdane-1,3-thiazole hybrids **99**, **100,** and **103**, **104** were obtained via the heterocyclization reaction of thiosemicarbazones **97**, **98**, and **101**, **102** with 2-bromoacetophenone [63].

The structures of all newly synthesized compounds were established based on spectral data (IR, ^1^H, ^13^C, and ^15^N NMR).

All newly synthesized compounds were tested in vitro for antifungal and antibacterial activity against pure cultures of five fungal species (*Aspergillus niger*, *Fusarium solani*, *Penicillium chrysogenum*, *P. frequentans*, *Alternaria alternata*) and Gram-negative (*Pseudomonas aeruginosa*) and Gram-positive bacteria (*Bacillus polymyxa*).

Compounds **78a**,**b** and **81a**,**b** possessed antifungal activity with minimal inhibitory concentrations (MIC = 0.25 and 0.19 μg/mL) comparable to that of the antifungal drug Caspofungin (MIC = 0.25 μg/mL) and also showed antibacterial activity (MIC = 4 and 3 μg/mL, respectively) comparable to that of the antibiotic Kanamycin (MIC = 4.0 μg/mL). Compound **97** possessed moderate antifungal activity with minimal inhibitory concentrations (MIC = 1.5 μg/mL) comparable to that of the antifungal drug Caspofungin (MIC = 0.2 μg/mL) and also showed significant antibacterial activity (MIC = 0.125 μg/mL), which was 24 times more active than the known antibiotic Kanamycin (MIC = 3.0 μg/mL).

Thus, for the first time, based on (−)-sclareolide **2**, efficient syntheses were achieved through its di-, tri-, tetra-, and pentanorlabdane derivatives, including by unconventional methods such as microwave irradiation, of a series of molecular hybrids containing thiosemicarbazone fragments or 1,3-thiazole units. Molecular hybrids **81a** and **81b** exhibited pronounced antimicrobial properties, while hybrid **97** demonstrated excellent selective antibacterial activity, which was patented [69].

### 2.6. Synthesis of Norlabdane-1,3-benzothiazole Hybrids

The chemistry of 1,3-benzothiazole and its 2-substituted derivatives has developed into a distinct field of research, driven by their high structural diversity [70,71,72,73,74]. The interest in compounds with 1,3-benzothiazole structural units is fueled by their biological properties, such as anticancer, antimicrobial, antioxidant, anti-inflammatory, antiviral, and other activities [75,76,77,78,79].

Little data is known from the specialized literature about natural and biologically active compounds that include a 1,3-benzothiazole moiety, and even less about terpene compounds [80,81,82].

Currently, researchers’ attention is focused on developing methods for the synthesis of substituted 1,3-benzothiazoles and their derivatives, using different types of catalysts to improve selectivity, purity, and product yield. Structure-activity relationship (SAR) studies particularly reveal that the structure of the substituent at the C_2_ carbon atom strongly influences the bioactivity of the compound. Various methods are known to lead to the formation of 2-substituted compounds with 1,3-benzothiazole structural units. The most commonly used synthetic method involves the cyclocondensation reaction of aromatic aldehydes or carboxylic acids, esters, acyl halides with *ortho*-aminophenol [74] or its disulfides [83].

Thioamides can be obtained by the conversion of amides using Lawesson’s reagent, and the course of the reaction and the yield depend on the structures of the substrates used [84].

As part of modern research focused on the development of new biologically active terpeno-heterocyclic compounds and as a logical complement to the research described in the previous subsections, our team has set a new goal: the synthesis of tetranorlabdane 1,3-benzothiazole hybrids. The data obtained will be presented below [85].

As a starting material for the synthesis of the mentioned compounds, (+)-sclareolide **2** was used, which was transformed into carboxylic acid **10** in three steps with a total yield of 89%. Carboxylic acids **8** and **11** were obtained from (+)-sclareolide **2** in five and six steps, with yields of 81% and 62%, respectively.

**Scheme 11:**
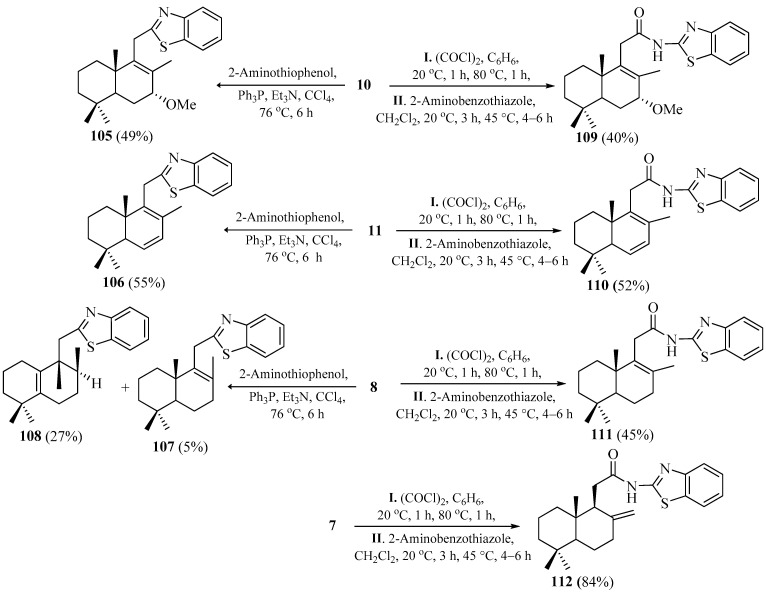
Synthesis of norlabdane-1,3-benzothiazole hybrids. Data from [85].

The one-pot decarboxylative cyclization reactions of acids **8**, **10**, and **11** with 2-aminothiophenol, promoted by triphenylphosphine and triethylamine [86], were carried out under reflux for four hours. After purification by column chromatography on silica gel, this afforded 2-homodrimenyl-1,3-benzothiazoles **105**–**108**, with yields as illustrated in Scheme 11.

It should be noted that, in the case of carboxylic acid **8**, surprisingly, in addition to the desired compound **107**, obtained with a yield of only 5%, the compound **108**, with an unexpected structure, was afforded as a major reaction product, with a total yield of 27%. The rearrangement of the carbon skeleton of compound **108** was confirmed by a shift in some signals in the ^1^H NMR spectrum compared to the starting acid **8**.

Next, a series of new *N*-homodrimenoyl-2-amino-1,3-benzothiazoles were prepared, starting from the intermediate carboxylic acids **7**, **8**, **10**, and **11** via their acyl chlorides generated in situ. It should be mentioned that the acid **7** was obtained from the commercially available (+)-sclareolide **2** in six steps, with an overall yield of 60%, according to the known method [22].

The desired *N*-substituted 2-amino-1,3-benzothiazoles **109**–**112** were obtained with yields between 40% and 84% through the acylation of 2-amino-1,3-benzothiazole with the mentioned sesquiterpene acyl chlorides under the stated conditions (Scheme 11). According to the NMR spectra, the hybrids involved both heterocyclic and terpene units, and their accurate masses were confirmed by a high-resolution mass spectrometry (HRMS) analysis.

All synthesized compounds were subjected to preliminary screening for their in vitro antifungal and antibacterial activities against pure cultures of fungal species *Aspergillus niger*, *Fusarium solani*, *Penicillium chrysogenum*, *Penicillium frequentans*, *Alternaria alternata*, as well as both Gram-positive *Bacillus* sp. and Gram-negative *Pseudomonas aeruginosa* bacterial strains. The obtained minimum inhibitory concentration (MIC) values revealed that compounds **105** and **110** possessed a high nonselective antifungal activity (MIC 0.094 and 0.25 μg/mL, respectively) in comparison with Caspofungin. Moreover, compounds **107**, **109**, and **110**, exhibited a promising antifungal activity at MICs in the range from 0.95 to 2 μg/mL, compared to the same standard. At the same time, compound **105** demonstrated high non-selective antibacterial activity (MIC 0.75) relative to the standard Kanamycin. Compounds **107**, **108**, and **110** exhibited a moderate antibacterial activity. As for compounds **109**, **111**, and **112**, they were biologically inactive.

In conclusion, short synthetic routes to a series of tetranorlabdane–1,3-benzothiazole hybrids were successfully developed starting from acids **7**, **8**, **10** and **11**. The highest antimicrobial activities were observed for homodrimane sesquiterpenoids containing 1,3-benzothiazole units, as well as for derivatives bearing the NCS fragment, which, when rigidly constrained by an additional ring, adopt a spatial configuration that mimics the steric and electronic features of the benzothiazole scaffold.

### 2.7. Synthesis of Norlabdane-1,3-benzimidazole Hybrids

1,3-Benzimidazole is a valuable structural element in the design of new drugs, due to its diverse biological activity, chemical stability, and ability to interact with a variety of biological targets, which makes it highly relevant to the pharmaceutical industry [87].

Synthetically, benzimidazoles are obtained through the condensation reaction of 1,2-phenylenediamine with aldehydes and carboxylic acids. Their derivatives exhibit low toxicity and high activity against many pathogenic strains, with minimal chances of resistance [88].

There are few studies describing the synthesis and biological evaluation of new hybrid heterocyclic structures. In one of them, molecular hybrids formed by 1,2,3-triazole and benzimidazole units are presented, which reveal significant antibacterial, antifungal, and cytotoxic activities [89]. Other authors reported the synthesis of a new series of hybrid molecules with benzimidazole-1,2,3-triazole units obtained in several steps, including microwave-assisted reactions. The synthesized hybrids showed a moderate inhibition of 30% in the Foa sporulation test [90]. To date, no molecular hybrids with norlabdne-benzimidazoles have been reported.

The aim of this research was to develop original methods for the synthesis of new terpeno–heterocyclic derivatives based on the readily available natural diterpenoid (−)-sclareol 1, and to design chiral natural molecules of potential interest to the pharmaceutical industry. In this context, we present the results of the synthesis of novel tetranorlabdanes bearing either 2-substituted 1,3-benzimidazole or *N*-substituted 2-amino-1,3-benzimidazole moieties, along with the evaluation of their antimicrobial activity [91].

**Scheme 12:**
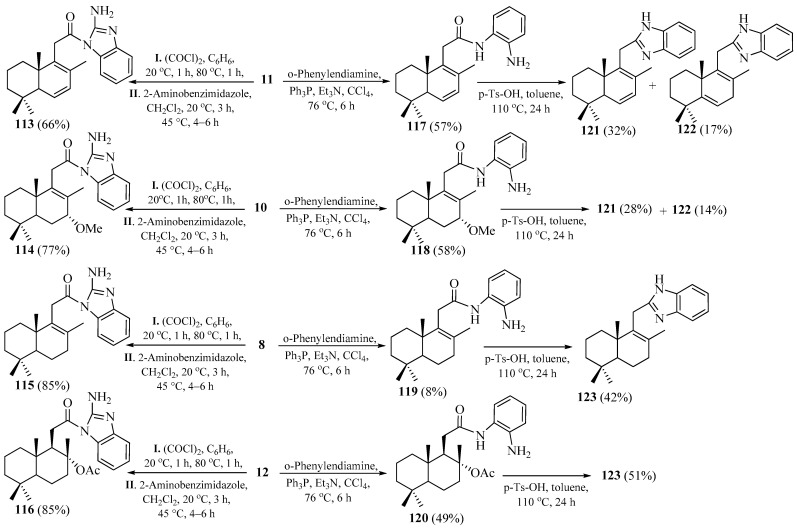
Synthesis of norlabdane-1,3-benzimidazole hybrids. Data from [91,96,97].

The title *N*-homodrimenoyl-2-amino-1,3-benzimidazoles was synthesized from intermediate carboxylic acids **8**, **10**, **11**, and **12**, via their in situ-generated acyl chlorides. The target compounds **113**, **114**, **115**, and **116** were obtained in yields ranging from 66% to 85% by acylating 2-amino-1,3-benzimidazole with the corresponding tetranorlabdane-derived acyl chlorides under the specified conditions [22] (Scheme 12).

NMR spectral analysis confirmed the presence of both heterocyclic and terpene moieties within the hybrid structures, while their molecular formulas were validated by high-resolution mass spectrometry (HRMS).

Multiple attempts to directly synthesize the target hybrid benzimidazoles via heterocyclization of acids **8**, **10**, **11**, and **12** with o-phenylenediamine in the presence of 4N HCl [92], glacial AcOH [93], or BF_3_·OEt_2_ [94] were unsuccessful. However, treatment with triphenylphosphine and triethylamine [86] afforded the corresponding monoacylated derivatives **117**–**120** in the yields indicated in Scheme 12. Additionally, diacylated derivatives were obtained in the reactions involving acids **8** and **11**.

The structures of the synthesized compounds were confirmed by the ^1^H, ^13^C, ^15^N, and 2D NMR spectroscopy, by the HRMS analysis, and finally, in the case of amide **120**, by the single-crystal X-ray diffraction (XRD).

Subsequently, the cyclodehydration of the resulting monoacylamides **117**–**120** was carried out using *p*-toluenesulfonic acid (*p*-TsOH) in toluene [95]. Monoacylamides **117** and **118** underwent cyclization to afford 2-substituted benzimidazoles **121** and **122** (Scheme 12). The formation of the doubly unsaturated benzimidazole **122** from amides **117** and **118** can be rationalized by the acid-promoted elimination of the C_7_-methoxy group from compound **118**, followed by proton abstraction at C_5_, which results in isomerization of the Δ^6,7^ double bond to Δ^5,6^. Under analogous conditions, monoacylamides **119** and **120** yielded the same benzimidazole 123 (Scheme 12). In the case of compound **120**, the generation of the Δ^8,9^ benzimidazole **123** derivative occurs via elimination of the C_8_-acetoxy group.

All synthesized compounds were subjected to preliminary screening for their in vitro antifungal and antibacterial activities [50] against pure cultures of the fungal species *Aspergillus niger*, *Fusarium solani*, *Penicillium chrysogenum*, *Penicillium frequentans*, and *Alternaria* bacterial strains. The obtained minimum inhibitory concentration (MIC) values revealed that compounds **115** and **120** possess the highest antifungal (MIC 0.064 and 0.05 μg/mL, respectively) and antibacterial (MIC 0.5 and 0.032 μg /mL, respectively) activities, followed by compound **113** (MIC 1.6 and 4.0 μg /mL, respectively), which is comparable to the standard’s activity. Compounds **114**, **116**, and **118** have shown moderate antifungal activity at MICs in a range from 0.80 to 1.16 μg /mL, and antibacterial activity at MICs in a range from 3.90 to 6.0 μg /mL, against the same standard.

In conclusion, it can be stated that a series of seven tetranorlabdane-1,3-benzimidazole hybrids were synthesized through the decarboxylative cyclization and condensation of the aforementioned acids or their acyl chlorides with *o*-phenylenediamine and 2-aminobenzimidazole, as well as the *p*-TsOH-mediated cyclodehydration of the mentioned monoacylamides. The hybrids were evaluated as antimicrobial agents, and six of them demonstrated high to moderate antifungal and antibacterial activities compared to those of the reference drugs. The activity of compounds **115** and **120** has also been patented [96,97].

## 3. Conclusions

The escalating prevalence of fungal and bacterial infections, compounded by the emergence of antimicrobial resistance, underscores the critical need for the development of novel molecular entities with enhanced antimicrobial efficacy. Natural products, and terpenoids in particular, have emerged as a prolific source of bioactive compounds due to their inherent biocompatibility, selective biological activity, and low toxicity profiles.

The summary of our publications from 2013 to the present shows that norlabdane hybrids bearing diazine, 1,2,4-triazole, 1,3,4-oxadiazole, 1,3,4-thiadiazole, 1,3-thiazole, 1,3-benzothiazole and 1,3-benzimidazole moieties represent valuable bioactive molecules for the design of new drugs.

The mentioned norlabdane-heterocyclic hybrids were synthesized from commercially available (+)-sclareolide **2**, derived from the labdane-type diterpenoid (−)-sclareol isolated from industrial *Salvia sclarea* L. waste via two main strategies: under normal conditions or conventional heating. In some cases, microwave heating was used to improve overall yields and reaction efficiency by reducing reaction times, solvents, and energy consumption, contributing to the greening of the synthetic process.

In the first case, coupling reactions of various terpene derivatives, such as carboxylic acids, acyl chlorides, or bromides, with selected azaheterocyclic compounds were preferred to form new C–N or C–C bonds and thus the desired hybrid molecules.

When heterocyclization reactions were applied, involving the cyclocondensation of key intermediates such as hydrazides, hydrazinecarbothioamides, or thiosemicarbazones, they led to the formation of heterocyclic rings integrated into the side chains or the B-ring of the terpene components.

The in vitro biological evaluation of these compounds against selected fungal strains and bacterial species revealed notable antimicrobial activity, ranging from moderate to pronounced and high, thereby validating their therapeutic potential. This promising biological activity exhibited by the synthesized hybrids highlights their potential as lead candidates for the development of new antimicrobial agents, thus contributing to both the advancement of medicinal chemistry and the sustainable utilization of renewable natural resources.

We believe that this progress in the field of molecular hybrids, especially terpeno-heterocyclic hybrids, will encourage their synthesis and more active exploration of their biological properties.

## Data Availability

The original contributions presented in this study are included in the article. Further inquiries can be directed to the corresponding author.

## Abbreviations

The following abbreviations are used in this manuscript:

CDI	1,1′-Carbonyl diimidazole
(COCl)_2_	Oxalyl chloride
CH_2_Cl_2_	Dichloromethane
CCl_4_	Tetrachloromethane
CNBr	Cyanogen bromide
C_6_H_5_COCH_2_Br	2-Bromoacetophenone
C_6_H_6_	Benzene
^13^C NMR	Carbon-13 nuclear magnetic resonance
^1^H NMR	Proton-1 nuclear magnetic resonance
^15^N NMR	Nitrogen-15 nuclear magnetic resonance
DCC	*N*,*N*′-Dicyclohexylcarbodiimide
DMAA	*N*,*N*-Dimethylacetamide
DMAP	Dimethylaminopyridine
DMF	Dimethylformamide
EDCI	1-Ethyl-3-(3-dimethylaminopropyl)carbodiimide
Et_3_N	Triethyl amine
EtOAc	Ethyl acetate
EtOH	Ethyl alcohol
Et_2_O	Diethyl ether
HeLa	Human cervical cells
HepG2	Human liver cancer cells
HCT116	Human colon cancer cells
HRMS(EI)	High-resolution mass spectrometry
K_2_CO_3_	Potassium carbonate
IR	Infrared spectroscopy
MeOH	Methanol
Me_2_CO	Acetone
MW	Microwave irradiation
MeSO_3_SiMe_3_	Trimethylsilyl methanesulfonate
MeCN	Acetonitrile
MIC	Minimum inhibitory concentration
KOH	Potassium hydroxide
NaHCO_3_	Sodium bicarbonate
NH_2_-NH_2_∙H_2_O	Hydrazine monohydrate
NH_2_NHCSNH_2_	Thiosemicarbazide
NH_2_NHCSNHC_6_H_5_	4-Phenylthiosemicarbazide
NMR	Nuclear magnetic resonance
PPh_3_	Triphenylphosphine
SC(NH_2_)_2_	Thiourea
R-NCS	Substituted aryl isothiocyanates
THF	Tetrahydrofuran
TMTD	Tetramethylthiuram disulfide
